# Preferences of Dairy Cattle for Supplemental Light-Emitting Diode Lighting in the Resting Area

**DOI:** 10.3390/ani12151894

**Published:** 2022-07-25

**Authors:** Angela M. Wilson, Tom C. Wright, John P. Cant, Vern R. Osborne

**Affiliations:** 1 Department of Animal Biosciences, University of Guelph, Guelph, ON N1G 2W1, Canada; awilso17@uoguelph.ca (A.M.W.); jcant@uoguelph.ca (J.P.C.); 2 Ontario Ministry of Agriculture, Food and Rural Affairs, 1 Stone Road, Guelph, ON N1G 4Y2, Canada; tom.wright@ontario.ca

**Keywords:** dairy cattle, lying behaviour, artificial light, wavelength, housing systems

## Abstract

**Simple Summary:**

The effects of light exposure on dairy cattle milk production are well known, whereas aspects of light quality and potential benefits on dairy production and health are currently undetermined. We developed a supplemental lighting system (i.e., in addition to existing natural and artificial light in the facility) to provide light to cows while lying down. This study assessed dairy cow preferences for three colours of light-emitting diode lighting in the free-stall area. Cows did not prefer lying down under any of the light-emitting diode light options provided. Our results suggest that short-term use of supplemental light-emitting diode lighting in the stall area was not avoided by cows and lays the groundwork to study various aspects of light-emitting diode light, including the quality (wavelength), intensity, and duration of exposure.

**Abstract:**

Light from the environment is important for vision and regulating various biological processes. Providing supplemental lighting in the stall area could allow for individually targeted or group-level control of light. This study aimed to determine whether dairy cattle had preferences for short-term exposure to white (full-spectrum) light-emitting diode (LED) light or no LED light, yellow-green or white LED light, and blue or white LED light in the stall area. In total, 14 lactating cows were housed in a free-stall pen with unrestricted access to 28 stalls. LED light was controlled separately for each side of the stall platform. Two combinations of light were tested per week, and each week consisted of three adaptation days and four treatment days. Lying behaviour and video data were recorded continuously using leg-mounted pedometers and cameras, respectively. Preference was assessed by the amount of time spent lying and the number of bouts under each light treatment. No differences occurred between treatments within each week for daily lying time and number of bouts. Similarly, no differences occurred between treatments within each time period. Further controlled studies of long-term exposure to different LED wavelengths and intensities are required to determine potential benefits on metabolic processes.

## 1. Introduction

Light affects how images are perceived and regulates physiological and behavioural processes that are governed by circadian and circannual rhythms [1,2,3]. In mammals, light is received through photoreceptor cells (rods, cones, and intrinsically photosensitive retinal ganglion cells (ipRCGs)) located in the retina of the eyes and this information is sent via vision (image forming) and non-vision neural pathways [1,2,4]. Light regulates circadian rhythms through the hypothalamic suprachiasmatic nucleus, which acts as the “master clock” and drives peripheral clocks throughout the body [4,5]. In particular, ipRGCs, which are optimally sensitive to shorter wavelengths (e.g., perceived as blue light; reviewed by Wahl et al. [6]), are vital in synchronising the circadian rhythm to the daily environmental light-dark cycles [7,8,9].

In dairy cattle, it is well established that the duration of light exposure, or light-dark cycle (photoperiod), impacts reproduction, growth, milk production, and health [10]. For example, daylength can be manipulated to increase milk production, and longer daylengths of 16 to 18 h during lactation increase milk production compared to natural photoperiods [10,11,12]. In contrast, there has been little work on the quality (wavelength) of light in dairy cattle. Blue light is known to suppress melatonin secretion in humans [6], dairy cows [13] and calves [14] and may decrease milk production [15]. Uncertainty still exists on how different wavelengths, whether individual or in combination, can be used to support production, growth, reproduction, and immune function in dairy cattle.

We developed an in-stall lighting system that is supplemental, i.e., used in addition to existing natural and facility lighting, with the overall aim of evaluating various artificial lighting spectra for the purposes of manipulating circannual rhythms to enhance production, reproduction, and growth. First, it was important to determine whether supplemental light-emitting diode (LED) light in the stall area targeted to cows while resting affected their preference for where they lie down. Weiguo and Phillips [16] used preference testing to determine whether dairy calves favoured supplemental light in a bedded area, and Götz et al. [17] investigated the preferences of young pigs for different LED lighting colour temperatures. To our knowledge, preferences for wavelengths have not been investigated in lactating dairy cattle. Although preference tests need to be interpreted with caution [18], they provide insight into important aspects of housing from the animal’s perspective, which has allowed for improvements in housing and handling [19].

The specific aim of this study was to determine the preferences of dairy cows for short-term exposure to white (full-spectrum) LED light or no LED light, yellow-green or white LED light, and blue or white LED light in the stall area. We chose yellow-green (564 nanometres (nm)) and blue (583 nm) light colours since they were near the wavelengths to which cattle have peak sensitivities [20]. A red LED light colour was not tested as cattle lack cones sensitive to red light [20].

## 2. Materials and Methods

### 2.1. Animals and Management

The experiment was conducted in late February and early March 2020 at the Elora Dairy Research and Innovation Centre (lat +43.64, long −80.40; University of Guelph, ON, Canada). Fourteen lactating Holstein dairy cows were used for the duration of the study. Cows were in their second lactation and were 225.7 ± 34.8 days in milk (DIM; mean ± SD) and had an average milk production of 35.8 ± 5.1 kg/d, body weight (BW) of 742.9 ± 40.9 kg, body condition score of 3.0 ± 0.4 on a 1-5 scale, and a gait score of 2.1 ± 0.2 points on a 1–5 scale [21].

Cows had ad libitum access to a total mixed ration (TMR) fed 2×/d at 1000 and 1430 h and fresh water at 2 self-filling water troughs per pen. Cows were milked 2×/day at 0430 and 1630 h in a 24-bale rotary parlour. Prior to the experiment, cows were housed at a stocking density of 100% or less in pens with 30 head-to-head free-stalls (EEZY Lunge Floor Mount Freestall, CANARM Ltd., Arthur, ON, Canada). The stall base was a rubber crumb-filled mattress with 2 layers of 2-cm polyurethane foam (Pasture Mat, Promat Ltd., Woodstock, ON, Canada). Stalls were cleaned 4×/day and bedded 2×/week using chopped straw bedding delivered to the centre of the stalls. The bedding was pulled back into the stalls as required during cleaning.

**Figure 1:**
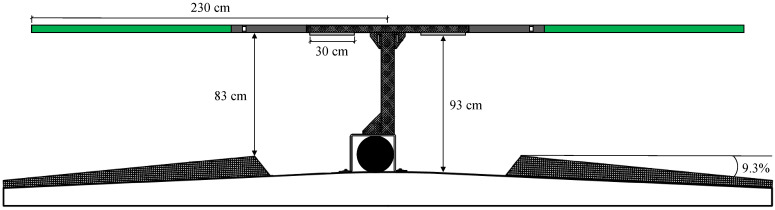
Sideview of the stall partitions containing light-emitting diode (LED) lights. The stall partitions were a novel design further modified from a previous experiment. LED lights were placed in an opening of the metal stall partitioning and secured using 3D-printed plastic clips. Foam (not visible) was placed inside the partitions between the LED light and metal to reduce the upward movement of the light. Wiring was contained in a 20-cm-diameter high-density polyethylene (HDPE) supply pipe (black circle in the figure) that ran along the centre of the stalls.

The design of the experimental stalls is described in Wilson et al. [22] and was further modified for this study (Figure 1). A metal extension was used to lengthen the horizontal part of the structural post. A flexible polyethylene tube was attached to the metal extension (130 cm long, 6 cm diameter; Hybrid Cow Stall material, Promat Ltd., Woodstock, ON, Canada). These experimental stalls did not have a neck rail or a brisket board. Instead, a deterrent strap was placed along the centre of the stalls at a height of 85 cm above the concrete, which prevented cows from walking through the stalls. In addition, a 20-cm-diameter high-density polyethylene (HDPE) supply pipe containing wiring for the lights ran along the centre of the stalls. Stalls had a slope of 9.3%.

Facility lights were on from 0400 to 2000 h and were fluorescent with a correlated colour temperature (CCT) of 4100 Kelvin (K; F32T8/TL841 PLUS ALTO HV, Philips Lighting, Amsterdam, Netherlands). Illumination in the experimental pen was 271 ± 29 (mean ± SD) lux (lx), recorded at 0500 h (before sunrise) on 2 separate days. In addition, 12 measurements were taken at standing cow eye height throughout the pen, and 28 measurements were taken at the approximate height of cows’ eyes standing and lying in the stalls (described in Table 1). The lux and wavelength of the facility lighting and treatment lighting were measured using a spectrometer (LI-180 Spectrometer, LI-COR Biosciences, Lincoln, NE, USA). Natural daylight entered the facility through side curtain walls along the feed alley. At the beginning of the experiment, the sunrise was at 0721 h and sunset at 1759 h (daylength 10.5 h). Natural daylength increased by 2.8–2.9 min/day; thus, at the end of the experiment, the sunrise was at 0649 h and sunset at 1815 h (daylength 11.4 h).

### 2.2. Experimental Setup

LED lights were installed in custom-manufactured free-stall partitions [22] at 95 cm above the concrete platform (Figure 1). The LED lights were situated above cows’ heads since, in mammals, light is received through photoceptor cells located in the retina of the eyes [4]. The structural post of the stall partition contained a cut-out section for the LED lights, which extended 2 cm below the partition and were held in place using 3D-printed plastic clips. Foam was positioned in the partition and used to buffer and limit upward movement of the light (e.g., if contacted by a cow).

The LED lights were 30 cm in length and contained red, green, blue (RGB), and white (2700 K) chips (LED Neon Light, Round Top RGBW, SGI Lighting, Halton Hills, Ontario, Canada). The LEDs contained a silicone filling and were protected against dust and temporary exposure to water (IP67 rating). The silicone coating also functioned as a lens and distributed light at a beam angle of 270 degrees.

**Figure 2:**
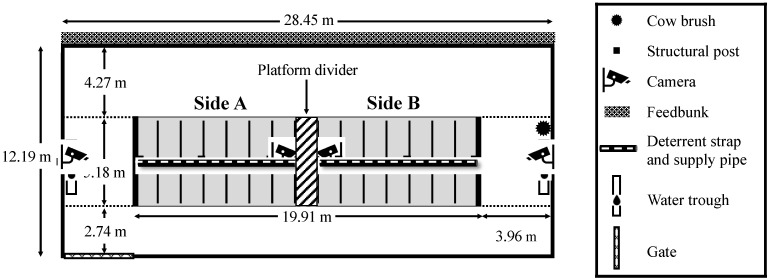
Layout of the experimental pen. The platform of a free-stall pen with 30 stalls was divided into 2 sides using an opaque divider. In total, 14 second lactation dairy cows had free choice of 28 modified free-stalls. Light treatments were programmed separately per side and switched sides halfway through each treatment period. Four cameras were positioned to view each side from two different angles. A deterrent strap prevented cows from walking through the head-to-head modified free-stalls. A supply pipe containing wiring for the LED lights ran along the base of the stall platform under the deterrent strap.

The platform of 30 head-to-head free-stalls was divided into 2 halves (Figure 2). The centre two stalls were blocked off with an opaque divider (high-density polyethylene, puck board) to prevent light transfer, resulting in a total of 28 stalls available to 14 cows. A cow brush (Vertical Cow Brush, Legend, Tillsonburg, ON, Canada) was located near the water trough on one side (side B) of the pen. Lights on each side of the platform were controlled separately. The colour, intensity, and timing of light were programmed using ESA Pro 2 software (Nicolaudie Architectural, Nicolaudie America Inc. Orlando, FL, USA). The desired wavelengths to test were converted into RGB values, which were then used to program the lights (Supplementary Materials, Table S1). A touch keypad was used to change settings manually and was located at the end of the platform above the stalls.

### 2.3. Treatments

**Figure A1:**
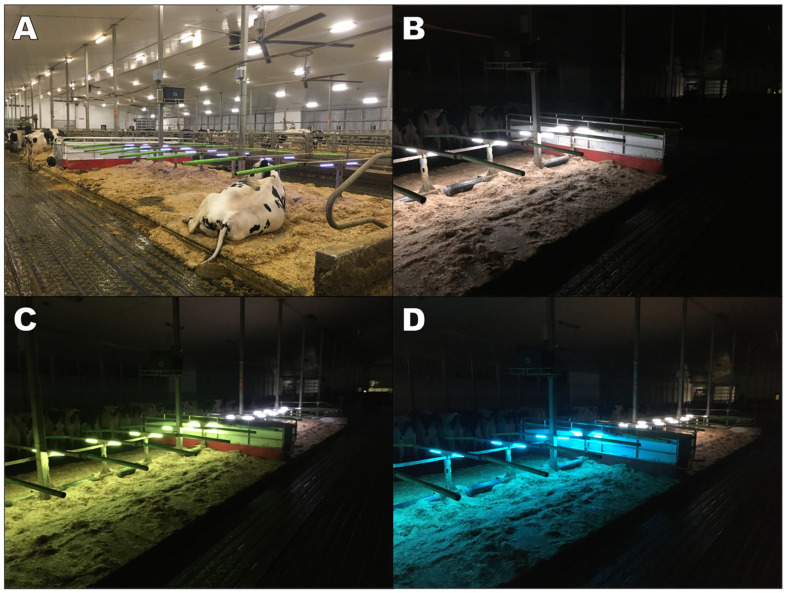
The experimental free-stall platform was divided into 2 halves using an opaque divider, resulting in 28 stalls available for 14 cows (**A**). The lights were programmed separately for each side. Pictures (**B**–**D**) show the light colours during the time period when only the LED lights were on (i.e., facility lighting was off). White light (380–780 nm) was tested against no LED light in week 1 (**B**). During week 2, yellow-green light (554 nm) was tested against white light (**C**) and during week 3, blue light (483 nm) was tested against white light (**D**).

Three different combinations of light were tested across three weeks and two light options were tested per week (Table 2). In week 1, cows had a choice between white light (full-spectrum, 380–780 nm, average 96 lx in the stall area) and no light (i.e., LED lights were off). In week 2, the two options were yellow-green light (564 nm, average 49 lx in the stall area) and white light (full-spectrum, 380–780 nm, average 66 lx in the stall area). In week 3, blue light (483 nm, average 35 lx in the stall area) and white light (full-spectrum, 380–780 nm, average 31 lx in the stall area) were tested. The white lights in weeks 2 and 3 were programmed for similar illuminance (i.e., lux) to the colours tested to minimise the possibility of cows choosing stalls based on the perceived brightness. Images of the three LED light colours are shown in Appendix A Figure A1. Light treatments were delivered at relatively low intensities (31 to 96 lx). In comparison, minimum lighting recommendations for dairy facilities are 100 lx in the stall area and 200 lx at the feedbunk during the day [23], and the American Society of Agricultural and Biological Engineers recommends 150 lx of illuminance throughout the barn [24]. However, these recommendations are for visual perception, rather than non-image forming vision roles, and the purpose of this supplemental LED lighting system was to influence biological processes through non-vision visual system pathways.

A forced-choice phase was not used due to the potential, but unknown, cumulative or carryover effects of light exposure, which could affect preferences. However, during the adaptation/washout periods of each week (days 1–3), all cows spent time lying down on each side of the platform at least once.

**Figure A2:**
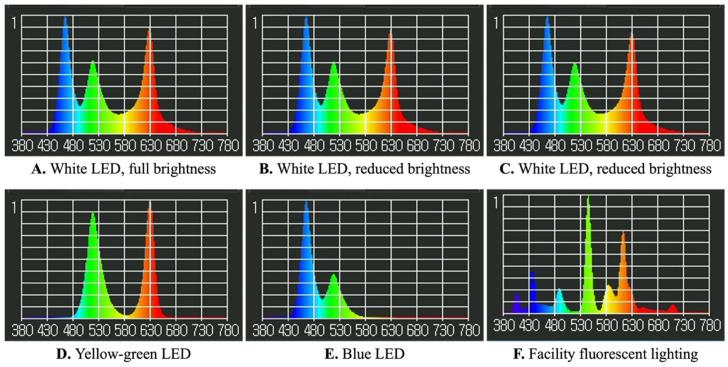
Spectral distributions produced by LED light treatments (**A**–**E**) and fluorescent facility lighting (**F**). Distributions shown are from one measurement from each light type.

Since preferences were based on lying behaviour, light measurements were taken at the stall level (Appendix A, Table A1 and Table A2, Figure A2). Lux and wavelength were measured in 4 places of every stall: (1) 2 cm directly under the light (in line with the partition), (2) 93 cm under the light (at the stall base; in line with the partition), (3) approximate cow eye height when lying down (in the centre of the stall between the partitions at 55 cm above the mattress and 61 cm from the front of the stall), and (4) the centre of the stall (between the partitions) 61 cm from the front of the stall and at the height of the mattress (11 cm above the concrete; depicted in Supplementary Materials, Figure S1). Wavelengths from LEDs bordering the opaque divider were not included as they varied considerably compared to all other measurements, probably due to the absorptivity and reflectivity of the divider material. LED light measurements were recorded during two time periods: (1) when only the LEDs were on and the rest of the facility was dark (2100 h), and (2) during the daytime when facility LED lights were on and there was daylight. Light measurements were collected either before or after the experimental period to avoid influencing cow behaviour. LED lights were wiped clean before measurements and once weekly throughout the experimental period. For all measurements, the spectrometer was held in a horizontal position with the sensor facing the ceiling to minimise variation resulting from the sensor direction. Spectral distribution of the LED lights was taken in a subset of stalls and fluorescent facility lighting spectral distributions were taken in three locations. Spectral distributions of the LED lights and facility lighting are shown in Appendix A Figure A2.

### 2.4. Experimental Design

Before the experiment, cows were housed together in a larger group of 30 cows and had no exposure to the LED light or preference test pen setup. During the experiment, cows had free access to all 28 stalls. Each week consisted of a 3-day adaptation (washout) phase followed by a 4-day treatment phase. Treatments were applied to both sides of the platform and switched sides after 2 days. Due to the limitations of animal availability and the practicality of conducting the experiment, the same animals were used across the three weeks. Thus, we assumed cows are independent of the treatments across weeks, i.e., that no carryover effects of light occurred across weeks.

Each treatment day was divided into three time periods to account for facility lighting and provide exposure to darkness (Table 2). The three time periods were: (1) dark period, from 0000 to 0400 h with no lights on during this time; (2) daytime period, from 0400 to 2000 h with LED light treatments and ambient lighting (facility lights, natural illumination from sunlight); and (3) LED light only period, from 2000 to 0000 h with only LED lights on. Facility lights were not on during the third period.

### 2.5. Data Collection

The lying time and number of bouts were recorded continuously using IceTag accelerometers (IceTag, IceRobotics, South Queensferry, UK) attached to the right hind leg above the fetlock joint. IceTag activation and data download were performed using the IceReader device and IceManager software (IceRobotics, South Queensferry, UK). IceManager software was used to generate summaries of all lying bouts for each cow, including the start date and time, end date and time, and duration.

Video recordings were analysed for all treatment days (i.e., four days per week). Stall location was determined using continuous video recording. In total, 4 2.8-mm fixed-lens high-definition IP dome cameras (DS-2CD2325FWD-I, HikVision, Hangzhou, China) were mounted on building structural posts at a height of approximately 3.2 m and positioned to capture the stalls from 2 different angles (i.e., 2 cameras per angle). Night recording was enabled by infrared illuminators in the cameras. Cameras were programmed to record at 30 frames/second and were powered by a switch (TPE-TG82g, TRENDnet, Torrace, CA, USA) connected to a computer (ThinkCentre M720 Tiny, Lenovo, Quarry Bay, Hong Kong). All system components, except the cameras, were housed in a shielded plastic box secured to a structural post above the experimental pen. Recordings were stored on an internal drive, which was archived 1×/day to an external 8 TB hard drive. Video footage was analysed using viewing software (XProtect Smart Client, Milestone, Brondby, Denmark) and cows were differentiated by their unique black and white markings. Lying bout data from each cow were matched to the video footage to determine the amount of lying time spent in each stall type.

BW was collected 2×/d and BCS was collected 1×/d upon exiting the parlour using an in-line scale (AWS100, DeLaval, Tumba, Botkyrka, Sweden) and a body condition scoring camera (DeLaval BCS, DeLaval, Tumba, Botkyrka, Sweden), respectively.

### 2.6. Statistical Analysis

Days 1 to 3 of each week were adaptation days and were not included in the main analysis. Preferences for LED lighting were examined in two ways: (1) on a daily basis and (2) within each time period to account for the potential effect of facility lighting. Lying bouts of less than or equal to 2 min in duration were removed from the data as they were considered erroneous [25]. These data were used to calculate the lying time and number of lying bouts of individual cows for each treatment per day and per time period. If a lying bout crossed over into another day (i.e., 2300 to 0100 h) or time period (i.e., 0330 to 0430 h), the bout was counted in the day or time period, respectively, in which it started. Lying time and number of bouts were summarised for each treatment by day for daily analysis and summarised by time period for time period analysis. An average was created for days 4 and 5 (light treatment 1 on side A, light treatment 2 on side B), and for days 6 and 7 (light treatments switched sides). These values were used in the analysis. Lying behaviour that occurred under the light treatments was compared within each week and not across weeks.

All data were analysed by a generalised linear mixed model ANOVA (PROC GLIMMIX) in SAS v.9.4 (SAS Institute Inc., Cary, NC, USA) with cow as the experimental unit. The covariance between repeated measures on cow was modelled using a compound symmetry covariance structure. Significance was declared at *p* ≤ 0.05 using an *F*-test. The model statement for daily measurements included terms for treatment within week, platform side, and cow:

(1) $$y_{ijkl}=\mu +TRMT{\left(WEEK\right)}_{ij}+SIDE_{k}+COW_{l}+\varepsilon_{ijkl}$$

where $y_{ijkl}$ is the observation on cow l with treatment i within week j on side k; μ is the overall mean; $TRMT{\left(WEEK\right)}_{ij}$ is the fixed effect of treatment i (i = 1, 2, 3, 4, 5, 6) within week j (j = 1, 2, 3);$\;SIDE_{k}$ is the fixed effect of side k; C is the random effect of subject cow l; and $\varepsilon_{ijkl}$ is the residual random error with mean 0 and variance $\sigma^{2}$. To examine whether preferences differed within time periods (i.e., dark period, daytime, and LED light only), the same model was used with the addition of terms for time period and the interaction of time period with treatment within week.

The assumption of an adequate washout phase was investigated by determining whether the daily lying time and number of bouts during the adaptation (washout) and treatment phases differed across weeks. Data were summarised per day per cow during the adaptation (days 1 to 3) and treatment (days 4 to 7) phases. The model statement included terms for phase, week, phase-by-week interaction, and cow:

(2) $$y_{ijk}=\mu +PHASE_{i}+WEEK_{J}+{\left(PHASE\times WEEK\right)}_{ij}+COW_{k}+\varepsilon_{ijk}$$

where $y_{ijk}$ is the observation on cow k in phase i in week j; μ is the overall mean; $PHASE_{i}$ is the fixed effect of phase i (i = adaptation, treatment); $WEEK_{j}$ is the fixed effect of week j (j = 1, 2, 3); ${\left(PHASE\times WEEK\right)}_{ij}$ is the interaction between fixed effects of phase i and week j; C is the random effect of subject cow k; and $\varepsilon_{ijk}$ is the residual random error with mean 0 and variance $\sigma^{2}$. Repeated measures of day within week and cow as the subject were modelled using a compound symmetry covariance structure.

Model assumptions (residuals with a random distribution, independence from treatment effects, homogeneity, normal distribution, and a mean of 0) were tested using the Shapiro–Wilk statistic and the UNIVARIATE procedure of SAS and were visually assessed using Q–Q plots, histograms, and box plots. Interactions with significance at *p* < 0.10 were examined using the SLICEDIFF option in LSMEANS with a Tukey’s adjustment. All outcome variables were Gaussian. Results are presented as least square means ± standard error of the mean.

Two cows were withdrawn from the study during week 2 due to health issues unrelated to treatment and their data for week 2 were removed. These cows were replaced with two new cows, also in their second lactation, at the beginning of week 3. A cow lost its IceTag on day 6 of week 3 and was missing data from days 6 and 7. During week 2, issues occurred with the light programming, resulting in data removal from days 5 and 7.

## 3. Results

### 3.1. Daily Lying Time and Number of Bouts

The assumption of an adequate washout phase was investigated by comparing daily lying behaviour data from the adaptation and treatment phases. The lying time and number of lying bouts did not differ between the adaptation and treatment phases across weeks (interaction between phase and week; *p* = 0.133, *p* = 0.706).

**Figure 3:**
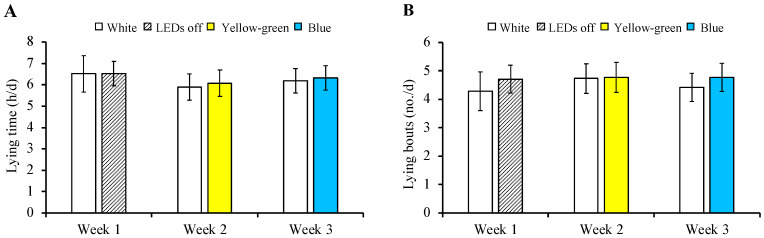
Least square means ± SE for daily lying time (**A**) and number of lying bouts (**B**) of 14 lactating dairy cows given free access to 2 different LED light options each week in a 3-week preference test. Weeks comprised 3 days of no light (adaptation) and 4 days of treatments. White light had a spectral distribution of 380–780 nm (full-spectrum), yellow-green light had a wavelength of 564 nm, and blue light had a wavelength of 483 nm. The white lights tested in weeks 2 and 3 were adjusted to a lux similar to that of the colours tested.

Daily, no differences occurred between treatments within each week for lying time (*p* = 0.980, Figure 3A). In week 1, cows spent 6.5 ± 0.71 h/d lying down under both white LED light and no supplemental light (LEDs off). In week 2, cows spent 6.0 ± 0.61 h/d lying down under both white and yellow-green LED lights. In week 3, cows spent 6.2 ± 0.58 h/d lying down under both white and blue LED lights.

Similarly, no differences occurred between treatments within each week for the number of lying bouts (*p* = 0.977, Figure 3B). In week 1, cows had 4.5 ± 0.58 bouts/d under both white LED light and no supplemental light (LEDs off). In week 2, cows had 4.8 ± 0.52 bouts/d under both white and yellow-green LED lights. In week 3, cows had 4.6 ± 0.50 bouts/d under both white and blue LED lights.

### 3.2. Time Period

**Figure 4:**
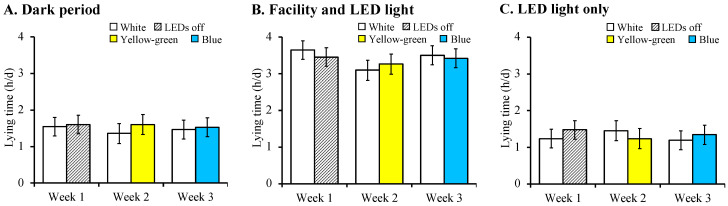
Least square means ± SE for lying time during the dark period (**A**), facility and LED lighting period (**B**), and LED light only period (**C**). Fourteen lactating dairy cows given free access to two different LED light options each week in a three-week preference test. White light had a spectral distribution of 380–780 nm (full-spectrum), yellow-green light had a wavelength of 564 nm, and blue light had a wavelength of 483 nm. The white lights tested in weeks 2 and 3 were adjusted to a lux similar to that of the colours tested. Weeks were comprised of 3 days of no light (adaptation) and 4 days of treatments. To account for the facility lighting, lying time was analysed in each time period. The dark period was from 0000 to 0400 h, the facility and LED light period was from 0400 to 2000 h, and the LED light only period was from 2000 to 0000 h.

**Figure 5:**
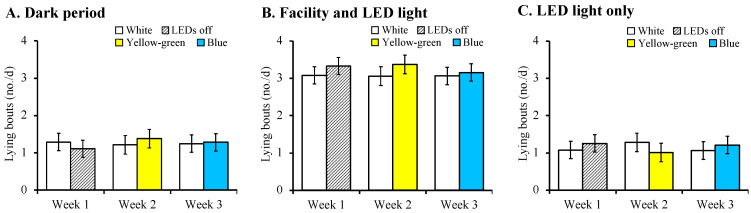
Least square means ± SE for number of lying bouts during the dark period (**A**), facility and LED lighting period (**B**), and LED light only period (**C**). Fourteen lactating dairy cows given free access to two different LED light options each week in a three-week preference test. White light had a spectral distribution of 380–780 nm (full-spectrum), yellow-green light had a wavelength of 564 nm, and blue light had a wavelength of 483 nm. The white lights tested in weeks 2 and 3 were adjusted to a lux similar to that of the colours tested. Weeks were comprised of 3 days of no light (adaptation) and 4 days of treatments. To account for the facility lighting, the number of lying bouts was analysed in each time period. The dark period was from 0000 to 0400 h, the facility and LED light period was from 0400 to 2000 h, and the LED light only period was from 2000 to 0000 h.

No differences between treatments within the time period occurred for the lying time (*p* = 0.979, Figure 4) and number of lying bouts (*p* = 0.976, Figure 5). Within each time period, cows spent a similar amount of time lying down and had a similar number of lying bouts in the supplemental light treatments within weeks.

### 3.3. Platform Side

The interactions between platform side and week were not significant for the lying time and number of lying bouts reported by day (*p* = 0.872 and *p* = 0.884, respectively) and by time period (*p* = 0.929 and *p* = 0.478, respectively).

## 4. Discussion

This study aimed to determine whether dairy cows had preferences for lying down under short-term exposure to white (full-spectrum) LED light or no LED light, white LED light or yellow-green (564 nm) LED light, and white LED light or blue (483 nm) LED lighting in the stall area. Cows did not show a preference for any of the light options provided indicated by similar lying times and numbers of bouts among treatments. Additionally, facility lighting did not affect preferences, as measures were similar within each time period.

Since cows did not show a preference for any of the light treatments, it is unclear whether cows were indifferent to the lights or unable to detect differences between them. Animals may be unable to differentiate between options in a preference test if those options are outside of the animals’ sensory and cognitive capabilities [19]. However, it is likely that the cows in our study could differentiate between the treatments provided in the first week (white light vs. LEDs off) since Weiguo and Phillips [16] found calves chose to spend more time lying down under supplemental light in the bedding area, compared to no supplemental light.

In weeks 2 and 3, we tested yellow-green and blue lights, respectively, against full-spectrum light with a similar illuminance. Cattle can differentiate between red and blue, and red- and green-coloured light, but have a limited ability to differentiate between blue and green [26]. Although no studies currently exist on whether cattle can differentiate between full-spectrum white light and single wavelengths, there is evidence to suggest that other dichromatic mammals can distinguish colour temperature [17,27]. Colour temperature is expressed in Kelvin, which indicates the relative colour of white light. Cooler colour temperatures are characterised by a higher degree of Kelvin (4000, 6500 K) and have a higher relative intensity of blue wavelengths compared to warmer colour temperatures (lower degree of Kelvin; 2500, 2700 K; [28]). Götz et al. [17] found that 4-week-old piglets could differentiate between LED light colour temperatures of 3000 (warmer; perceived as reddish by humans) and 6500 K (cooler; bluish). Pigs are dichromatic, similar to cattle. Cattle have cones that are maximally sensitive at 554 and 455 nm [20] and pigs have cones sensitive to 556 and 439 nm [29]. Furthermore, Paronis et al. [27] found male laboratory mice spent more time in cages with warm fluorescent lighting (2500 K) compared to cool fluorescent lighting (4000 K). Cattle, pigs, and mice all have cone photoreceptors sensitive to short and medium wavelengths. Since differences in colour temperature are the result of differences in wavelengths and there is evidence that pigs and mice can distinguish between colour temperatures, it is, therefore, likely that cattle are also capable of differentiating between the single wavelengths (blue, yellow-green) and full-spectrum (white) light that were tested in our study.

The LED lights in our study were delivered at a relatively low intensity. The average illuminance of the LEDs tested in the lying area ranged from 31 to 96 lx as measured when the facility was dark, i.e., not including the lux of the facility lighting. The intensity of light appears to have a dose-dependent effect on melatonin production. Both Murphy et al. [13] and Lawson and Kennedy [30] observed that all light intensities decreased the melatonin concentration within the first few hours of exposure to darkness. However, Murphy et al. [13] found that 225 lx of blue light was required to suppress plasma melatonin concentrations. In contrast, Lawson and Kennedy [30] found that 400 lx of fluorescent light was required for sustained melatonin inhibition. In the current study, it is probable that cows could detect the different light treatments at least during the time period when only the LED lights were on (facility lights off, no natural daylight), as horses, also dichromats, were able to detect colour differences at as low as 0.08 lx [31]. However, since we observed no preferences for LED lights regardless of whether the facility lights were on, it is unknown whether the cows could detect the presence of LEDs when accompanied by the facility lights (i.e., whether the LEDs were “washed out” by the facility lighting or natural daylight). Consequently, using a higher lux could have caused different results in our study.

We used a spectrometer to take measurements of lux in various areas of the stall area. The spectrometer was facing toward the ceiling (horizontal) for all measurements to ensure consistency. However, an important consideration is that this sensor placement is not at the same angle or position that cows’ eyes face; therefore, the lux we report may be less than the lux the cows perceive. Ideally, light should be measured and quantified to correspond with how light is perceived by cows’ eyes [32].

Our study investigated preferences for short-term exposure to LED light wavelengths and does not indicate how cows’ preferences may change across a week- or month-long light exposure. Götz et al. [17] found that when pigs were given a choice between 3000 and 6500 K LED light, the pigs favoured the 3000 K colour temperature in the first week. This preference decreased in the third week, and in the fifth week, pigs did not have a preference for either colour temperature [17]. The observed effects of light exposure could also be shortly after exposure (e.g., 2 h), as seen in the plasma melatonin concentrations of dairy cows exposed to blue light (465 nm; [13]) and in humans exposed to blue-enriched light before sleeping [33]. Alternatively, responses may take a couple of weeks to be evident. According to Dahl and Peticlerk [34], the increase in milk production in response to the long-day photoperiod develops gradually and becomes significant after three to four weeks. This is likely due to the circadian rhythm entraining to a new lighting schedule, as biological clocks gradually adapt to a new daylength (proposed by Murphy et al. [13]). In our study, cows were behaviourally entrained to an LD16:8 photoperiod before and during the study (i.e., facility lights were turned off at 2000 h). As the time period with LED light only occurred outside the normal photoperiod, a longer duration of treatment lighting may be required to elicit changes in lying behaviour. Thus, cows likely maintained their prior lying behaviour rhythms throughout the preference tests. Additionally, the 4 h of LED light only would likely have been a time when cows were resting in stalls, given that milking occurred at 0430 and 1630 h, and feeding at 1000 and 1430 h [35,36].

An additional consideration of our study is that LED light without the influence of facility or natural lighting could only be tested during 2000 and 0000 h, which resulted in an LD20:4 light cycle (i.e., 4 h of complete darkness). As daylength drives the circadian rhythm, this experimental setup could influence cow circadian rhythms and may not be desirable over a longer testing period or outside experimental conditions.

There are some limitations of our study. First, the experimental design assumes that a 3-day washout (adaptation) phase between treatments is sufficient to mitigate any cumulative effects of 4 days of light exposure. As we were relying on responses from the immediate perception of light in the cows’ environment, rather than the responses from non-vision pathways (e.g., cortisol levels, milk production), it is likely that the washout phase was adequate. Furthermore, Murphy et al. [13] observed no carryover effects when cows were exposed to 8 h of blue light in one eye. Regardless of the lux used, plasma melatonin concentrations did not differ between nights with no treatment applied [13]. In our study, lying behaviour in the adaptation phases did not differ across weeks or from the treatment phases. Thus, we are confident that the washout phase was adequate for the duration and type of treatments tested. A second limitation is that a relatively small number of animals were used over a short period of time. The results of our study are for short-term light exposure and additional research on long-term exposure is needed. A further limitation is the lack of a restriction phase where animals are forced to experience both options, which may help reduce fear and reluctance toward new environments (e.g., [37]). However, a restriction phase to each light type in our study was avoided due to the potential, but unknown, for cumulative or carryover effects from light exposure. Moreover, all light treatments were provided on both sides of the stall platform to minimise the impact of location on preference.

This study was designed to determine whether providing supplement LED lighting in the stall area affected cows’ preferences for where they lie down. Our supplemental lighting system allows for customisable control of the wavelength (colour), intensity, and timing of light. Thus, it could be used for a wide variety of applications in dairy cow facilities, including individual- or group-level targeted light control. Regarding future directions of this work, it is necessary to study the long-term controlled exposure to light in the stall area. It would be interesting to program the LED lights to mimic a spectrum closer to that of natural daylight and determine whether this can further support milk production, health, or other biological responses over long-day photoperiods. The wavelengths of natural daylength have a higher proportion of blue and yellow wavelengths early in the morning that are important for setting the circadian rhythm [38]. The circadian rhythm is not only sensitive to the intensity of light but also the spectral distribution of light [38,39]. Finally, when using lights close to cows’ eyes, high-quality LED lighting should be used to minimise or avoid the potential adverse health effects resulting from the “flicker” or stroboscopic effects of LED lights [40,41]. The stroboscopic effect has been reported and studied in humans, and animals may also be affected.

## 5. Conclusions

The purpose of this current study was to determine the preferences of dairy cows for short-term exposure to different LED wavelengths (full-spectrum, yellow-green, and blue) using a supplemental lighting system. Similar lying times and numbers of bouts occurred, suggesting that under the conditions of this study, cows did not have preferences for the light options tested. In addition, whether cows were in the dark, under facility and LED lighting, or LED lighting only did not affect preference. The findings suggest that the use of LED lighting in the stall area, short term, is not avoided by cows. This study lays the groundwork for future research into the use of supplemental LED lighting to influence metabolic processes and animal behaviour, such as affective state.

## Figures and Tables

**Table 1:** Facility light (fluorescent lighting) distribution in the experimental pen.

		Lux (lx)		Wavelength (nm) ^1^	
Location	Observations	Mean	SD	Mean	SD
Alleyways and crossovers, standing height ^1^	12	284	27	576	0
Stalls ^2^, standing height	28	280	31	577	1
Stalls ^2^, lying height	28	262	25	577	1

Note 1: all measurements were taken using a spectrometer with the sensor facing the ceiling (i.e., horizontal) to minimise variation resulting from the sensor position. Note 2: measurements were recorded at 0500 h (before sunrise) on two separate days. Note 3: the measurement heights are cows’ approximate eye height when standing and lying. ^1^ 122 cm above the pen floor. ^2^ Measurements were taken in the centre of the stall (between the two partitions) at 61 cm from the front of the stall, and 133 and 66 cm above the concrete platform for standing and lying, respectively.

**Table 2:** Layout of the lighting schedule by time period and treatments across weeks.

	Week		
Time Period	1	2	3
	White LED vs. LED light off	White LED ^1^ vs. yellow-green LED	White LED ^1^ vs. blue LED
0000 h–0400 h	All lights off (dark period)		
0400 h–2000 h	Treatment LEDs on, facility lights ^2^ on, ambient daylight		
2000 h–2400 h	LED lights only		

Note: Two LED light options were tested on days 3 to 7 each week. Cows had free access to lie down under either type. ^1^ White LED light in weeks 2 and 3 was programmed for similar lux to the colours tested to minimise the possibility of cows choosing stalls based on the perceived brightness. ^2^ Fluorescent lights.

## Data Availability

Data available upon reasonable request from corresponding author.

## Supplementary Materials

The following supporting information can be downloaded at: https://www.mdpi.com/article/10.3390/ani12151894/s1, Figure S1: Diagram illustrating cows standing and lying in stalls with LED lights. Measurements of light illuminance (lux) and wavelength were taken in four areas of each stall and are indicated by the red stars: (1) 2 cm directly under the light (in line with the partition), (2) 93 cm under the light (at the stall base; in line with the partition), (3) approximate cow eye height when lying down (in the centre of the stall between the partitions at 55 cm above the mattress and 61 cm from the front of the stall), and (4) the centre of the stall (between the partitions) 61 cm from the front of the stall and at the height of the mattress (11 cm above the concrete); Table S1: The intended wavelengths, the programmed red, green, and blue (RGB) values, and the measured wavelength of LED light colours used.

## Appendix A

**Table A1:** Descriptive statistics of LED light illuminance (lux) and wavelength measured during two time periods: (1) when the facility was dark (no external light), and (2) during the daylight hours with LED and facility lights on. The average (±SD) of measurements taken in four places ^1^ per stall in all stalls (*n* = 28) are provided.

	Lux (lx)		Wavelength (nm) ^2^	
Lighting	Mean	SD	Mean	SD
**White (week 1)**				
Dark, LEDs only	96	95	472	2
Daylight	394	114	576	2
**White (week 2) ^2^**				
Dark, LEDs only	66	69	471	2
Daylight	417	101	577	1
**Yellow-green (week 2)**				
Dark, LEDs only	49	46	564	0
Daylight	418	99	574	2
**White (week 3) ^2^**				
Dark, LEDs only	31	33	463	4
Daylight	418	86	576	1
**Blue (week 3)**				
Dark, LEDs only	35	35	483	0
Daylight	406	89	555	36

Note 1: all measurements were taken using a spectrometer with the sensor facing the ceiling (i.e., horizontal) to minimise variation resulting from the sensor position. Note 2: daylight conditions were cloudy with minimal sun and measurements were taken between 1000 and 1100 h on one day. ^1^ Wavelength measurements at the divider were not included. Stalls at the end of each platform were measured in the stall centre only since lights were not installed in the end partitions. ^2^ Wavelength reported is the most prominent wavelength in each measurement and does not reflect the distributions of wavelengths.

**Table A2:** Descriptive statistics of LED light illuminance (lux) and wavelength measured when the facility was dark (no external light). Measurements were taken in four places ^1^ per stall in all stalls (*n* = 28).

	Lux (lx)		Wavelength (nm) ^2^	
Lighting	Mean	SD	Mean	SD
**White (week 1)**				
Directly under light	257	37	474	1
Under light, stall base	49	3	472	2
Centre of stall, eye height	35	6	472	1
Centre of stall, mattress height	43	8	471	1
**White (week 2) ^3^**				
Directly under light	184	21	472	1
Under light, stall base	33	2	471	2
Centre of stall, eye height	20	4	471	1
Centre of stall, mattress height	29	5	471	1
**Yellow-green (week 2)**				
Directly under light	127	22	564	0
Under light, stall base	26	2	565	1
Centre of stall, eye height	20	3	564	0
Centre of stall, mattress height	23	5	565	0
**White (week 3) ^3^**				
Directly under light	85	20	466	3
Under light, stall base	15	1	463	3
Centre of stall, eye height	10	2	461	4
Centre of stall, mattress height	14	3	461	3
**Blue (week 3)**				
Directly under light	93	13	483	0
Under light, stall base	17	1	483	0
Centre of stall, eye height	13	2	483	0
Centre of stall, mattress height	16	3	483	0

Note: all measurements were taken using a spectrometer with the sensor facing the ceiling (i.e., horizontal) to minimise variation resulting from the sensor position. ^1^ Wavelength measurements at the divider were not included. Stalls at the end of each platform were measured in the stall centre only since lights were not installed in the end partitions. ^2^ Wavelength reported is the most prominent wavelength in each measurement and does not reflect the distributions of wavelengths. ^3^ Illuminance of white lights in weeks 2 and 3 were reduced to be similar to the illuminance of yellow-green and blue lights, respectively.
