# Supplementary material for: Preferences of Dairy Cattle for Supplemental Light-Emitting Diode Lighting in the Resting Area
Source: Animals (Basel). 2022 Jul 25;12(15):1894. doi: 10.3390/ani12151894 (PMC9331357; doi:10.3390/ani12151894)
Supplement: Supplementary file 1 [file animals-12-01894-s001.zip › animals-1816738-supplementary.pdf]

animals MDPI

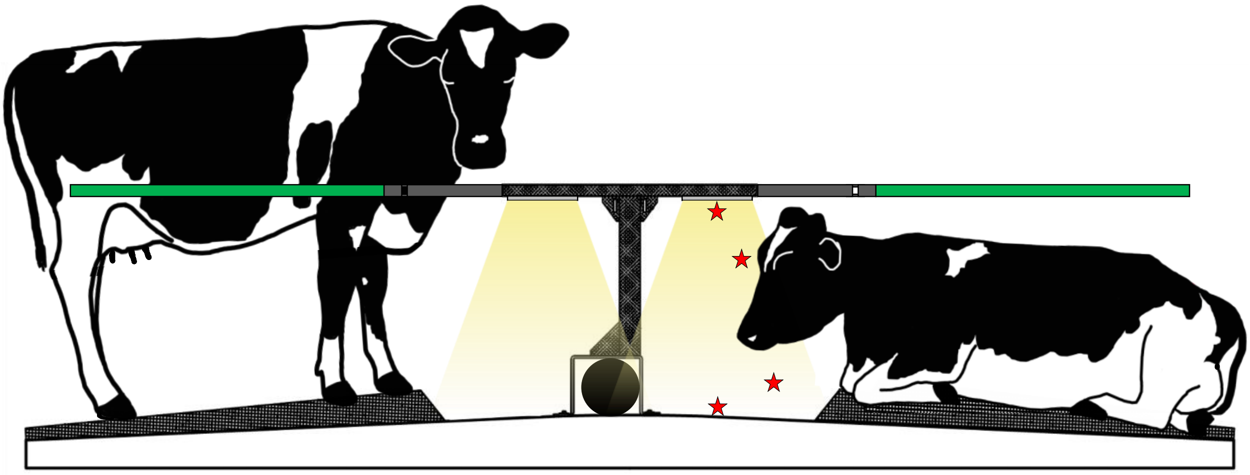

Figure S1. Diagram illustrating cows standing and lying in stalls with LED lights. Measurements of light illuminance (lux) and wavelength were taken in four areas of each stall and are indicated by the red stars: (1) 2 cm directly under the light (in line with the partition), (2) 93 cm under the light (at the stall base; in line with the partition), (3) approximate cow eye height when lying down (in the centre of the stall between the partitions at 55 cm above the mattress and 61 cm from the front of the stall), and (4) the centre of the stall (between the partitions) 61 cm from the front of the stall and at the height of the mattress (11 cm above the concrete).

Table S1. The intended wavelengths, the programmed red, green, and blue (RGB) values, and the measured wavelength of LED light colours used.

LED Light Colour	Intended Wavelength (nm)	Programmed RGB Values			Measured Wavelength (nm) [1]
		Red	Green	Blue	
White [2]	380–780	255	255	255	469 [3]
Yellow-green	570	225	255	0	564
Blue	475	0	192	255	483

[1] Wavelengths were measured using a spectrometer when the facility was dark. [2] White lights during weeks 1, 2, and 3 had the same wavelength programmed. [3] White lights were full-spectrum (380–780 nm) and the wavelength reported is the most prominent wavelength in the spectral distribution.
